# Predominant Single Stable VpmaV Expression in Strain GM139 and Major Differences with *Mycoplasma agalactiae* Type Strain PG2

**DOI:** 10.3390/ani12030265

**Published:** 2022-01-21

**Authors:** Maysa Santos Barbosa, Joachim Spergser, Lucas Miranda Marques, Jorge Timenetsky, Renate Rosengarten, Rohini Chopra-Dewasthaly

**Affiliations:** 1 Institute of Microbiology, Department of Pathobiology, University of Veterinary Medicine, 1210 Vienna, Austria; maysabarbosa_06@hotmail.com (M.S.B.); joachim.spergser@vetmeduni.ac.at (J.S.); Renate.Rosengarten@vetmeduni.ac.at (R.R.); 2 Department of Microbiology, Institute of Biomedical Science, University of São Paulo, Sao Paulo 05508-000, Brazil; lmirandamarques@gmail.com (L.M.M.); joti@usp.br (J.T.); 3 Multidisciplinary Institute of Health, Federal University of Bahia, Vitoria da Conquista 45029-094, Brazil

**Keywords:** mycoplasmas, surface lipoproteins, antigenic phase variation, immune evasion, MALDI-ToF MS

## Abstract

**Simple Summary:**

MALDI-ToF MS analysis demonstrates major differences between *Mycoplasma agalactiae* type strain PG2 and strain GM139. Evaluation of the Vpma (variable proteins of *M. agalactiae*) phenotypic profiles of these two strains reveals that unlike PG2, GM139 expresses a single Vpma, namely VpmaV, and without any visible phase variation. Although the presence of only one Vpma seems to be sufficient for the pathogenesis of the GM139 strain, the absence of phase variation can compromise immune system evasion and spread to other anatomical sites in the host.

**Abstract:**

Although mycoplasmas have a reduced genome and no cell wall, they have important mechanisms for the antigenic variation in surface lipoproteins that modulate their interactions with the host. *Mycoplasma agalactiae*, the main etiological agent of contagious agalactia, has a multigene family involved in the high-frequency phase variation in surface lipoproteins called variable proteins of *M. agalactiae* (Vpmas). The Vpma lipoproteins are involved in the immune evasion, colonization, dissemination, and persistence of *M. agalactiae* in the host. In this paper, we evaluate the Vpma phenotypic profiles of two different strains of *M. agalactiae,* namely, GM139 and the type strain PG2, to assess possible correlations between Vpma phase variability and the geographic localization, animal origin, and pathogenicity of these two strains. Using monospecific Vpma antibodies against individual Vpmas in immunoblots, we demonstrate that, unlike PG2, which expresses six Vpma proteins with high-frequency phase variation, colonies of GM139 predominantly express VpmaV and do not exhibit any sectoring phenotype for any Vpma. Since VpmaV is one of the most important Vpmas for cell adhesion and invasion, its predominant sole expression in GM139 without high-frequency variation may be the basis of the differential pathogenicity of GM139 and PG2. Additionally, MALDI-ToF MS analysis also demonstrates significant differences between these two strains and their relatedness with other *M. agalactiae* strains.

## 1. Introduction

*M. agalactiae* is the main etiological agent of the contagious agalactia (CA) syndrome notifiable to the OIE. CA affects small ruminants in several countries of the world, but has a far greater impact in the Mediterranean countries [1] with a prevalence of up to 66.7% among herds in an endemic area of Spain [2]. In the United States, *M. agalactiae* is associated with mastitis and arthritis in goats [3], and only sporadic cases of its isolation were reported in the recent years [4]. In South America, this pathogen has been identified in goat herds causing mastitis, polyarthritis, conjunctivitis, fever, anorexia, high-morbidity and mortality [5,6,7,8,9,10]. It has been isolated from milk [6,7,8,9,11], semen [11], and nasal swabs [10]. Congenital infections in goats were also described [12]. Asymptomatic forms of the disease in goats also occurred, and it was observed that the etiologic agent is able to be disseminated into the environment for twelve months to eight years in the absence of clinical signs [13,14].

*M. agalactiae* strain GM139 was isolated from the mastitic milk of a goat in the USA [15], whereas the pathogenic type strain PG2 was isolated from an infected sheep in Spain by Dr. C. Lopez of Madrid [16]. Experimental infections with PG2 caused reduced milk production, excretion of mycoplasmas in milk, as well as pathogen spread and survival in lymph nodes [17,18,19]. On the other hand, infections caused by GM139 seem to be restricted to the udder, although there are no more detailed data concerning the infection caused by this isolate [15].

Although mycoplasmas have reduced genomes (580–1350 Kbp), many of their genes are affected by the diversification of their cell surfaces [20]. The use of variable genes organized in families allows for the generation of an extensive repertoire of antigenic variation, maximizing the potential of a limited genome [21].

*Mycoplasma hyorhinis* was the first mycoplasma where phenotypic switching of diverse surface lipoproteins was described in the year 1990 [22]. The gene family encoding these phase- and size-variable lipoproteins (Vlps) was also shown to be involved in the adhesion to host cells [23,24]. The lipoprotein VlhA, a hemagglutinin in *M. synoviae*, is notable as one of the most well-studied phase-variable proteins in mycoplasmas [25]. There are several other membrane-variable lipoproteins in mycoplasmas, such as the pMGA in *M. gallisepticum* [26], Vmm in *M. mycoides* subsp. *mycoides* [27], and Vaa in *M. hominis* [28]. Similarly, *M. bovis*, a very close phylogenetic relative of *M. agalactiae*, exhibits the phase variation in the Vsp family of surface lipoproteins, which is considered to be homologous to the Vpmas of *M. agalactiae* and provides specific structural and antigenic characteristics to the pathogen [29,30].

So far, only one multigene family in *M. agalactiae* involved in the high-frequency DNA rearrangements of surface lipoproteins has been described, and it is known as the *vpma* [31] or the *avg* locus [32]. This sophisticated system of high-frequency antigenic phase variation affects the expression (phase variation) and/or the structure of genes in the multigene family [17,31,33] mediated by the Xer1 site-specific recombinase [34,35] or by non-Xer1 recombination under the selective pressure induced by the host’s immune response [17]. In the *M. agalactiae* type strain PG2, six genes are present in the *vpma* locus (*vpmaV, vpmaX, vpmaY, vpmaU, vpmaW,* and *vpmaZ*), together with one *xer1* recombinase gene [36]. Although 23 CDS related to the *vpma* locus were identified in the field strain 5632 at two different genomic loci, the majority of the other analyzed strains (89 out of 92 strains) possess a *vpma* profile similar to PG2, and the duplication of the *vpma* locus was considered a rare event [37,38]. Analyses of the *vpma* genes of strain 5632 showed that the 23 *vpma* genes are present on two different loci. Locus I contains 16 *vpma* genes, whereas Locus II contains 7 *vpma* genes that are duplicated on Locus I. Out of these 23 genes, *vpmaW* and *vpmaX* are allelic versions of *vpmas* in PG2, and 7 others, namely, *vpmaA*, -*B*, -*C*, -*D1*, -*D2*, -*E*, and -*F1*, are duplicated. Additionally, alleles of *vpmaD* (*vpmaD1* and -*D2*) and *vpmaF* (*vpmaF1* and -*F2*) varying in the C-terminal repeats are also present [37]. Therefore, the 5632 strain has 12 different *vpma* genes compared to PG2 [37]. However, information about the *vpma* genes, their structure or organization, and their expression in strain GM139 is missing from the literature. 

In the *M. agalactiae* type strain PG2 [16,39], Vpma surface proteins (VpmaV, VpmaU, VpmaX, VpmaW, VpmaY, and VpmaZ) as well as their antigenic phase variations, were shown to be involved in host immune evasion [33], pathogen dissemination [18], and *M. agalactiae* adhesion/invasion to host cells [40]. Due to the importance of the roles played by these phase-variable lipoproteins, in the current study, we evaluated the Vpma phenotypic profiles of the type strain PG2 [16] and the GM139 strain [15] to assess the correlations of the antigenic variability between these strains from different geographical localizations and animal origins, and any possible implications of the differences in disease characteristics.

## 2. Materials and Methods

### 2.1. Bacterial Strains, Culture Conditions 

*Mycoplasma agalactiae* strain GM139 [15] isolated from mastitic goat milk (CA, USA) and type strain PG2 [16] isolated from an infected sheep (Madrid, Spain) were grown at 37 °C in SP4 medium for 48 h or an SP4 agar for 4–5 days supplemented with 500 U/mL of penicillin, pyruvate (0.5%, *w*/*v*), and phenol red (0.005%, *w*/*v*), as described previously [41]. The strains were submitted to DNA extraction [21] and *M. agalactiae* species-specific PCR [42] before further analysis.

### 2.2. Western and Colony Immunoblots

Vpma expression on the surface of *M. agalactiae* colonies was evaluated by the colony immunoblots described previously [34]. Briefly, nitrocellulose membrane discs of 0.22 µm (Amersham™ Protran™, Cytiva, Marlborough, MA, USA) were placed on freshly grown mycoplasma colonies on the surface of agar plates for 5 min before being detached and dried at room temperature (RT). The membranes were rinsed three times in TBS (10 mM Tris and 154 mM NaCl, at pH 7.4) and incubated overnight at 4 °C in the six different Vpma-specific antisera (dilution of each Vpma antisera was performed as described by Chopra-Dewasthaly et al. [34]). After overnight incubation, the nitrocellulose membranes were washed three times for 10 min each with TBS and then incubated with secondary antibody anti-rabbit IgG conjugated to horseradish peroxidase (1:2000, DakoCytomation, Glostrup, Denmark). After three washes (10 min each), the colony blots were developed with 4-chloro-1-naphthol (Bio-Rad Laboratories, Vienna, Austria) and hydrogen peroxide. Colonies of *M. gallisepticum* and *M. ovipneumoniae* were used as negative controls. The reaction was stopped by washing the blots in water and counterstained using Ponceau S staining (Carl Roth, Vienna, Austria). The blots were photographed using a Nikon SMZ-U stereomicroscope (Nikon Corp., Tokyo, Japan).

The Vpma phenotypes of the two *M. agalactiae* strains were also checked by Western blot analysis of their Triton X-114 (Sigma, Vienna, Austria) fractions as described elsewhere [34]. Briefly, samples were treated at 95 °C for 5 min under reducing conditions and subjected to SDS-PAGE. Separated proteins were transferred to nitrocellulose membranes (Amersham™ Protran™, Cytiva, Marlborough, MA, USA) using a blotting buffer (48 mM Tris, 39 mM glycine, 0.037% SDS, and 20% methanol) and incubated separately in the six Vpma-specific antisera. Immunoreactive proteins were stained and visualized as described above for colony immunoblots. *M. gallisepticum* Triton X-114 extracts were used as negative controls.

### 2.3. MALDI-ToF MS Analysis

Matrix-assisted laser desorption ionisation-time of flight mass spectrometry (MALDI-ToF MS) of the late-exponential-phase broth cultures of *M. agalactiae* GM139 and of type strain PG2 was performed on a Microflex LT Biotyper (Bruker Daltonics, Bremen, Germany) configured with Bruker flexControl 3.4 software and compared against an in-house mycoplasma reference database containing more than 900 main spectrum profiles (MSPs), as described previously [43].

For protein extraction and the generation of spectra, 1 mL of GM139 and PG2 culture was grown for 3 days and centrifuged at 20,000× *g* for 7 min. The pellet was washed with 100 µL of HPLC-grade water (Sigma-Aldrich, Vienna, Austria) and centrifuged at 20,000× *g* for 5 min. The pellet was then subjected to extraction with equal volumes of 70% formic acid and acetonitrile. After centrifugation at 20,000× *g* for 2 min, the protein extract was spotted onto a 96-target polished steel plate (Bruker Daltonics, Bremen, Germany), air-dried, and overlaid with α-cyano-4-hydroxycinnamic acid matrix solution. MSPs were created and a score-oriented dendrogram was constructed based on the correlation distance measure with the average linkage algorithm (Bruker Daltonics), determined by comparing MSPs to the reference spectra of *M. agalactiae* strains integrated in the in-house database. Mass spectra were checked visually for characteristic strain-discriminating peaks using FlexAnalysis 3.4 software (Bruker Daltonics, Bremen, Germany).

## 3. Results and Discussion

**Figure 1:**
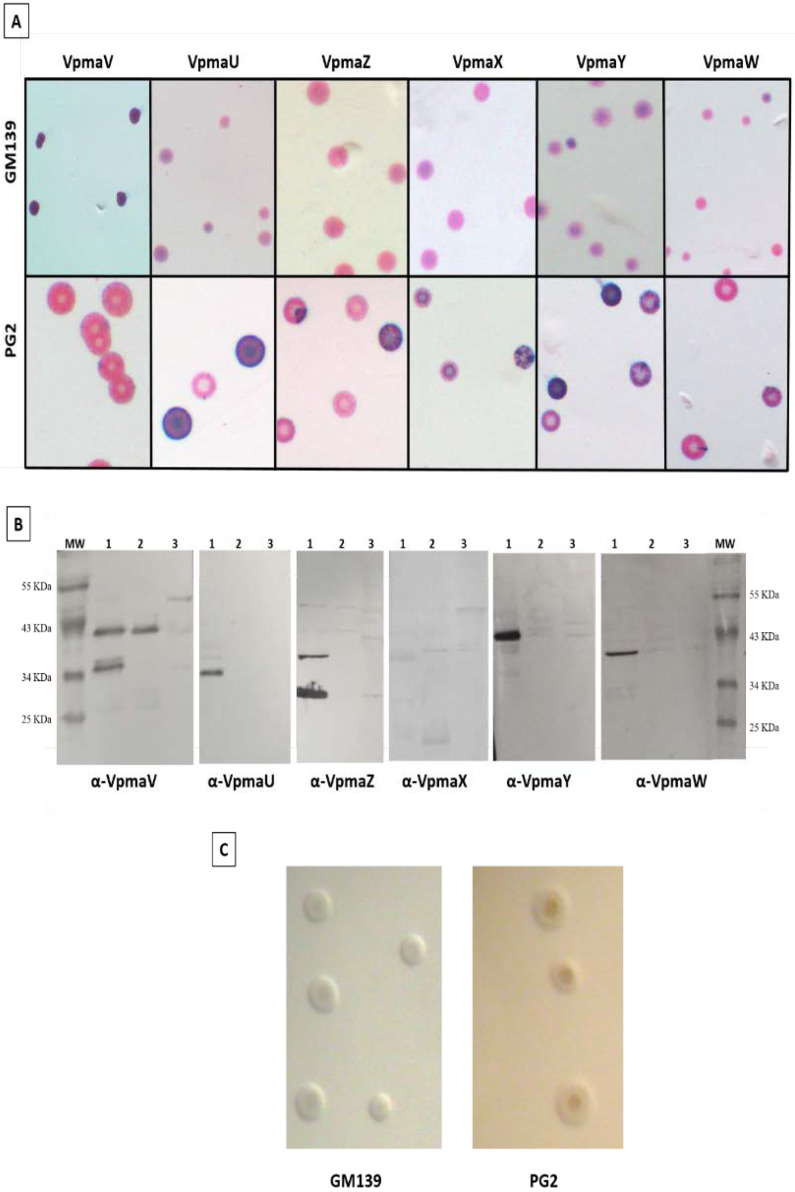
Vpma expression profiles of *Mycoplasma agalactiae* strains PG2 and GM139. (**A**) Colony immunoblot analyses of strain GM139 (upper row) and PG2 (lower row) using the six Vpma-specific antibodies recognizing specific surface-exposed epitopes. The purple color corresponds to specific antibody staining and indicates the expression of Vpmas on the surface of the mycoplasma cells, whereas the pink color corresponds to the non-specific protein counter-staining with Ponceau. (**B**) Western blot analysis demonstrating recognition of Vpmas by mono-specific Vpma antisera after extraction of lipoprotein fractions by Triton X-114. Lane 1: *M. agalactiae* strain PG2; Lane 2: *M. agalactiae* strain GM139; Lane 3: *M. gallisepticum* (negative control); MW: molecular weight protein marker. (**C**) Representative colonies of GM139 and PG2 strains after growth on SP4 agar at 37 °C. All micrographs were made using a Nikon SMZ-U stereomicroscope.

Colony immunoblot analyses of *M agalactiae* type strain PG2 and strain GM139 were performed to evaluate their Vpma expression profiles. Figure 1 demonstrates the differences in the Vpma profiles of strains PG2 and GM139 of *M. agalactiae*. The PG2 strain shows sectors corresponding to the high-frequency phase variation in the expression of all six Vpmas, amongst which VpmaV is the least expressed (Figure 1A) [34]. On the contrary, GM139 does not exhibit a sectoring phenotype for any of the six tested Vpmas and shows a predominant expression of the VpmaV phenotype. The Western blot results in Figure 1B further demonstrate the expression of only VpmaV in strain GM139 and the absence of all other five Vpma products in PG2 (Original whole Western blot figures see Supplementary Figures S1 and S2). Although the sole presence of VpmaV in the GM139 strain could imply a compromised host immune evasion due to a lack of Vpma phase variation, it highlights the significance of this Vpma protein, which appears to be sufficient for causing infection of the mycoplasma. In a previous study demonstrating Vpmas as major cytadhesins of *M. agalactiae,* phase-locked mutant V (PLM V) expressing a single stable VpmaV product exhibited the highest adhesion rate to HeLa cells, as well as sheep mammary epithelial (MEC) and stromal (MSC) cells [40]. Interestingly, VpmaV carries many cytoadhesion motifs that are involved in the host adhesion of the homologous Vsp proteins of *M. bovis* [44]. The 5′ untranslated region and the N-terminal-coding region of *vpma* genes bear 90% homology to the same regions of the *vspA*-coding sequence in *M. bovis* [32], which has been suggested to be involved in host cytoadhesion [36,44]. Although initially regarded as a predominantly extracellular pathogen, in vitro and in vivo infection experiments in recent years clearly demonstrated that *M. agalactiae* is capable of invading non-phagocytic cells such as the primary sheep mammary and uterine cells [45], HeLa, bovine endometrial cells (BEND), and buffalo lung fibroblast (BLF) cells, thus causing systemic spread inside the host [46]. VpmaU is the protein with the least invasive capacity and VpmaV indicated the highest rate of invasion in HeLa cells [40]. The expression of VpmaV in strain GM139 may indicate a level of invasiveness similar to that of PG2 clonal variants expressing VpmaV.

The lack of expression of any *vpma* genes other than *vpmaV* in the strain GM139, i.e., *vpmaX, vpmaY, vpmaW, vpmaU,* and *vpmaZ*, as observed in the PG2 type strain, cannot be explained currently, as nothing is known concerning the genetic organization of the *vpma* locus in this strain. GM139 genome sequencing and thorough genetic analysis are underway and are anticipated to fill in these gaps in our knowledge. A previous study revealed the enrichment of VpmaX and VpmaW expressors both during experimental and natural *M. agalactiae* infections [17], thereby indicating the importance of and the selective advantage conferred by these phenotypes inside of the host. The lack of these Vpmas in the GM 139 strain is likely to affect its disease characteristics, especially considering the visible absence of sectoring or high-frequency phase variation among the Vpmas. The latter was shown to be necessary for the expression of Vpma immune evasion proteins in order to escape the immune response and to survive and persist inside the immunocompetent host [17,18,33].

Both the GM139 and the PG2 strains have similar morphological characteristics when grown on SP4 agar (Figure 1C), despite different geographical and host origins [15,16], and different *vpma* profiles (Figure 1A,B). During experimental subcutaneous infections in the shoulders of lactating ewes, PG2 led to hypogalactia with a reduction in milk production, the excretion of mycoplasmas in milk, and the dissemination and survival of the pathogen in the lymph nodes, even in infections at low concentrations (10^3^ CFU/mL), demonstrating the ability of PG2 to establish a systemic infection [19], as was also witnessed in the experimental conjunctival and intramammary route infections of PG2 [17,18]. On the other hand, infections caused by GM139 seemed to be restricted to the udder, even when large amounts of mycoplasmas were recovered from the mammary secretions, although there are no more detailed data on the infection caused by this isolate [15].

Molecular analysis of GM139 demonstrated a completely different hybridization profile compared to those of PG2 and some other isolates from Israel from both goat and sheep with keratoconjunctivitis, arthritis, or contagiousa agalactia [32]. In another molecular typing study of Spanish *M. agalactiae* samples (*n* = 410), genomic homogeneity characterizing the circulation of a single endemic clonal population was revealed using PFGE (pulsed-field gel electrophoresis) analysis. In this study, PG2 shared the same pulsotype with the isolate Teramo (Italy) and the isolates from Valladolid (Spain) in the PFGE analysis. PG2 also clustered with the Teramo isolate, together with the strain 10123 (USA) during multilocus sequence typing (MLST) analysis [47]. However, a previous molecular typing study indicated an unexpected genetic diversity in an endemic area of Spain, whereby PG2 demonstrated the same pulsetype as bulk tank milk isolates with similar Vpma profiles. However, isolates from semen samples did not show hybridization bands for *vpmaU, vpmaV, vpmaY*, or *vpmaZ*, demonstrating that they had a different *vpma* repertoire than isolates from milk samples and from joints (5632 strain) [48]. Furthermore, previous studies evaluating the expression of surface epitopes of *M. agalactiae* isolates from 10 different countries also demonstrated high phenotypic diversification, which is also related to the geographical origin of the isolates [49].

**Figure 2:**
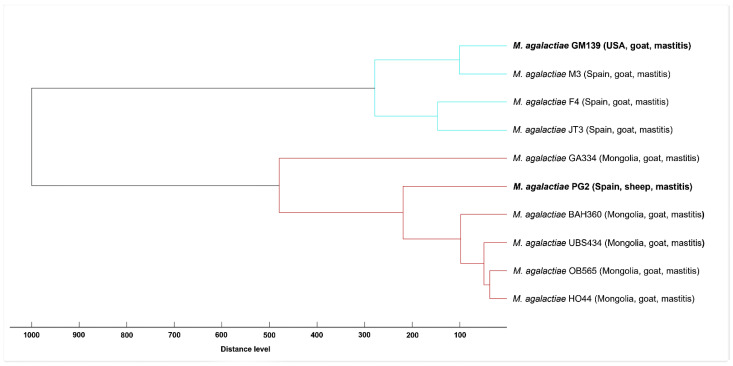
Score-oriented dendrogram based on distances between MSPs created from *M. agalactiae* GM139 and PG2 compared to MSPs of *M. agalactiae* strains from Spain and Mongolia integrated in the in-house mycoplasma library.

We used MALDI-ToF MS analysis to compare the MSPs of GM139 and of PG2 strains against the reference spectra of eight selected *M. agalactiae* strains integrated into the in-house database. In the constructed dendrogram, two clearly separated clusters are evident. *M. agalactiae* GM139 groups together with *M. agalactiae* strains from Spain, whereas *M. agalactiae* PG2 forms a cluster with Mongolian *M. agalactiae* strains (Figure 2), which further corroborates the subtyping capability of MALDI-ToF MS for *M. agalactiae* [50]. Spectral concordance between GM139 and PG2 expressed by a log score value of 1.75 demonstrated major differences between these two strains, whereas comparison between GM139 and strain M3 (isolated in Spain) resulted in a high log score of 2.35.

**Figure 3:**
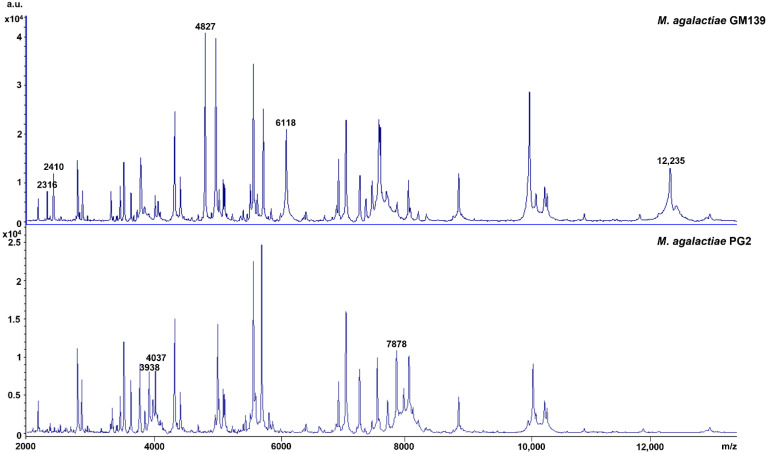
Peptide mass fingerprints of *M. agalactiae* GM139 and PG2 highlighting strain-specific peaks.

In addition, eight strain-specific peaks were found in the peptide mass fingerprints (PMF) that discriminate *M. agalactiae* GM139 from *M. agalactiae* PG2, with peaks at *m/z* 2316, 2410, 4827, 6118, and 12,235 Da present in GM139 and absent in PG2, and 3938, 4037, and 7878 Da present in PG2 and absent in GM139 (Figure 3). PMFs in the mass range of 2–20 kDa are considered to represent mainly ribosomal proteins along with a few housekeeping proteins. However, further in-depth analyses of several clonal variants are needed before correlating any of the respective peaks with specific Vpma phenotypes. 

## 4. Conclusions

In conclusion, the Vpma expression profile of *M. agalactiae* strain GM139 is peculiar, as it expresses a single Vpma, namely VpmaV, without any visible phase variation. Although VpmaV has the greatest adhesion capacity and invasiveness compared to all other Vpmas [40], these important pathogenicity characteristics are seemingly sufficient for the pathogenesis of the *M. agalactiae* strain GM139 strain, as this strain is known to cause mastitis in goats [15]. Yet, the absence of other Vpmas and, especially, the lack of Vpma phase variation might be a factor compromising its immune evasion characteristics and its systemic spread during an infection. Further studies, including molecular analyses, should be carried out on a large number of more recent *M. agalactiae* field isolates to check for the presence of different *vpma* genes and their expression. In addition, the genomic sequencing of more isolates of this mycoplasma species is expected to provide further information concerning these observations.

## Data Availability

All relevant data are already present in this manuscript or in the Supplementary Materials.

## Supplementary Materials

The following supporting information can be downloaded at: https://www.mdpi.com/article/10.3390/ani12030265/s1, Figure S1: Whole Western blot analysis demonstrating recognition of Vpmas by mono-specific Vpma antisera after extraction of lipoprotein fractions by Triton X-114. Lane 1: *M. agalactiae* strain PG2; Lane 2: *M. agalactiae* strain GM139; Lane 3: *M. gallisepticum* (negative control); MW: molecular weight protein marker. Figure S2: Densitometric analyses using ImageJ software. Western blotting band densities of Vpmas presented in Figure 1B. Band densities were quantified as a ratio to Vpma from PG2 strain.
