# Supplementary material for: Predominant Single Stable VpmaV Expression in Strain GM139 and Major Differences with Mycoplasma agalactiae Type Strain PG2
Source: Animals (Basel). 2022 Jan 21;12(3):265. doi: 10.3390/ani12030265 (PMC8833448; doi:10.3390/ani12030265)
Supplement: Supplementary file 1 [file animals-12-00265-s001.zip › animals-1510701-supplementary.pdf]

Supplementary Materials

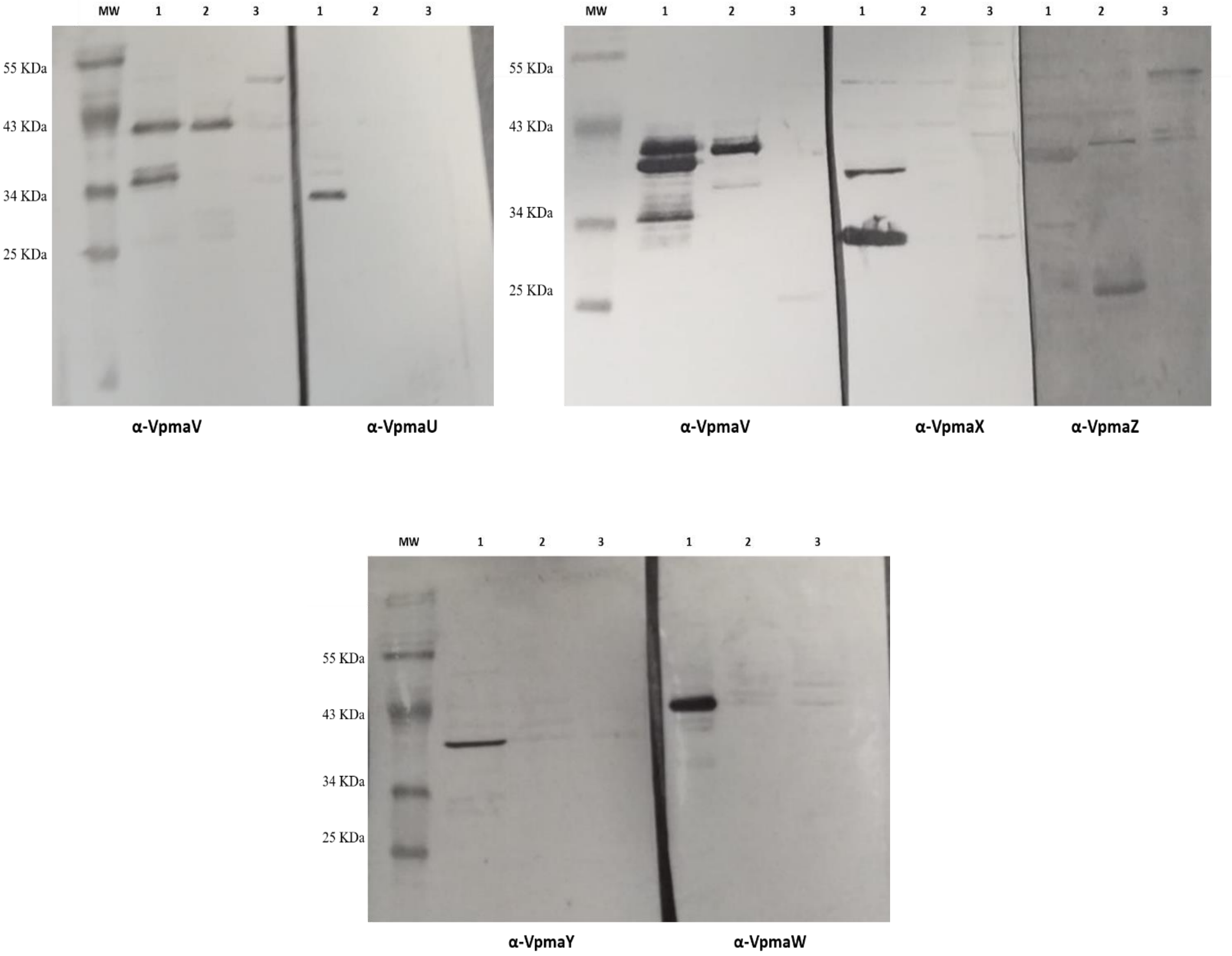

Figure S1. Whole Western blot analysis demonstrating recognition of Vpmas by mono-specific Vpma antisera after extraction of lipoprotein fractions by Triton X-114. Lane 1: *M. agalactiae* strain PG2; Lane 2: *M. agalactiae* strain GM139; Lane 3: *M. gallisepticum* (negative control); MW: molecular weight protein marker.

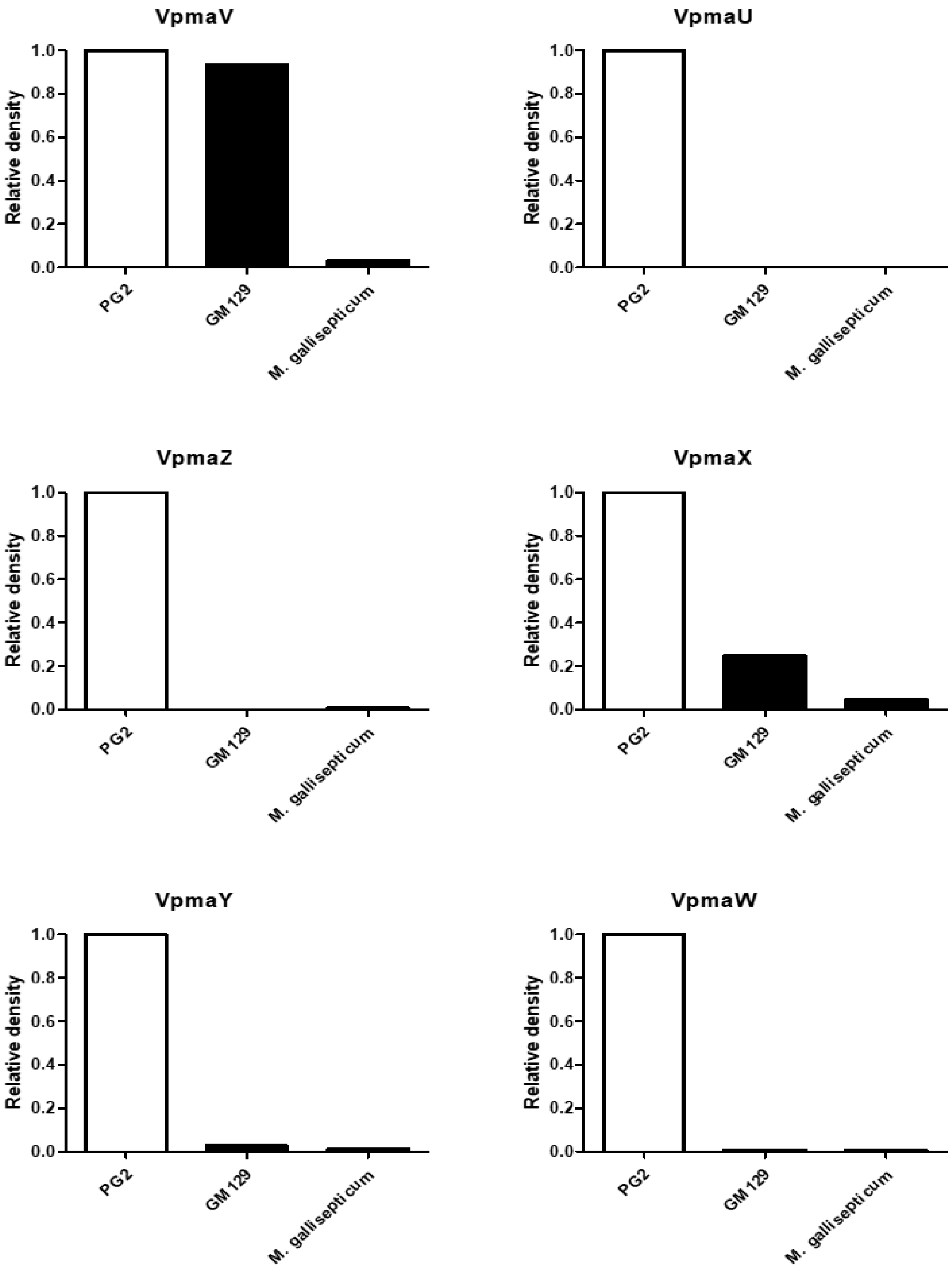

Figure S2. Densitometric analyses using ImageJ software. Western blotting band densities of Vpmas presented in Figure 1B. Each band densities were quantified as a ratio to Vpma from PG2 strain.
